# Characterisation and prion transmission study in mice with genetic reduction of sporadic Creutzfeldt-Jakob disease risk gene *Stx6*

**DOI:** 10.1016/j.nbd.2023.106363

**Published:** 2023-11-22

**Authors:** Emma Jones, Elizabeth Hill, Jacqueline Linehan, Tamsin Nazari, Adam Caulder, Gemma F. Codner, Marie Hutchison, Matthew Mackenzie, Michael Farmer, Thomas Coysh, Michael Wiggins De Oliveira, Huda Al-Doujaily, Malin Sandberg, Emmanuelle Viré, Thomas J. Cunningham, Emmanuel A. Asante, Sebastian Brandner, John Collinge, Simon Mead

**Affiliations:** a Medical Research Council Prion Unit at University College London (UCL), UCL Institute of Prion Diseases, London W1W 7FF, UK; b Mary Lyon Centre at MRC Harwell, Harwell Campus, Oxfordshire OX11 0RD, UK; c Division of Neuropathology and Department of Neurodegenerative Disease, UCL Queen Square Institute of Neurology, Queen Square, London WC1N 3BG, UK

**Keywords:** Prion disease, Creutzfeldt-Jakob disease, SNARE, Syntaxin-6, Incubation period

## Abstract

Sporadic Creutzfeldt-Jakob disease (sCJD), the most common human prion disease, is thought to occur when the cellular prion protein (PrP^C^) spontaneously misfolds and assembles into prion fibrils, culminating in fatal neurodegeneration. In a genome-wide association study of sCJD, we recently identified risk variants in and around the gene *STX6*, with evidence to suggest a causal increase of *STX6* expression in disease-relevant brain regions. *STX6* encodes syntaxin-6, a SNARE protein primarily involved in early endosome to *trans*-Golgi network retrograde transport. Here we developed and characterised a mouse model with genetic depletion of *Stx6* and investigated a causal role of *Stx6* expression in mouse prion disease through a classical prion transmission study, assessing the impact of homozygous and heterozygous syntaxin-6 knockout on disease incubation periods and prion-related neuropathology. Following inoculation with RML prions, incubation periods in *Stx6*^−/−^ and *Stx6*^+/−^ mice differed by 12 days relative to wildtype. Similarly, in *Stx6*^−/−^ mice, disease incubation periods following inoculation with ME7 prions also differed by 12 days. Histopathological analysis revealed a modest increase in astrogliosis in ME7-inoculated *Stx6*^−/−^ animals and a variable effect of *Stx6* expression on microglia activation, however no differences in neuronal loss, spongiform change or PrP deposition were observed at endpoint. Importantly, *Stx6*^−/−^ mice are viable and fertile with no gross impairments on a range of neurological, biochemical, histological and skeletal structure tests. Our results provide some support for a pathological role of *Stx6* expression in prion disease, which warrants further investigation in the context of prion disease but also other neurodegenerative diseases considering syntaxin-6 appears to have pleiotropic risk effects in progressive supranuclear palsy and Alzheimer’s disease.

## 1 Introduction

Prion diseases are transmissible neurodegenerative conditions of humans and animals with no established treatments. The infectious agent of prion diseases, or prion, comprises amyloid assemblies of misfolded host prion protein in a parallel in-register beta sheet conformation (Kraus et al., 2021; Manka et al., 2022). Sporadic Creutzfeldt-Jakob disease (sCJD), the most common human prion disease, invariably results in fatal neurodegeneration. sCJD is thought to result from the spontaneous formation of prions in the body by misfolding andaggregation of the host-encoded prion protein (PrP^C^), triggering a self-perpetuating cycle whereby prion fibrils elongate by recruitment of cellular PrP with subsequent fission producing more infectious particles. Variation in the *PRNP* gene encoding PrP^C^ is a key genetic determinant of sCJD risk and phenotype, however there is now robust evidence for two non-*PRNP* genetic risk factors identified through a genome-wide association study. This study found a genetic region including *STX6* as a novel risk locus in human sCJD (Jones et al., 2020). Interestingly the *STX6* locus has also been implicated in other neurodegenerative diseases, with the same genetic variants being associated with progressive supranuclear palsy (PSP) (Höglinger et al., 2011; Ferrari et al., 2014) and increased syntaxin-6 protein expression being causally associated with Alzheimer’s disease (AD) (Wingo et al., 2021; Yu et al., 2021), indicating this protein may play a pleiotropic role in neurodegeneration.

*STX6* encodes syntaxin-6, a member of the SNARE (soluble N-ethylmaleimide-sensitive factor attachment protein receptor) protein family, which is involved in the final step of membrane fusion during vesicle transport. Syntaxin-6 has been shown to be primarily involved in vesicle fusion during retrograde transport between early endosomes and the *trans*-Golgi network (Bock et al., 1996; Bock et al., 1997). Analysis of expression quantitative trait loci (eQTLs) at this site identified *cis-*acting variants which increase *STX6* expression in disease-relevant brain regions, particularly the putamen and caudate nuclei, which are commonly found to be abnormal in diagnostic MRI brain studies in sCJD (Zerr et al., 2009). These and other gene prioritisation analyses identify *STX6* as the causal gene driving the increased sCJD risk at this locus.

Experimental inoculation of mice with prions faithfully recapitulates the neuropathological hallmarks seen in human prion diseases, notably the propagation and deposition of PrP aggregates, spongiform degeneration, neuronal loss and widespread glial cell and immune activation (Chandler, 1961; Aguzzi et al., 2013). Furthermore, it has been long recognised that prions exist as conformationally distinct strains (Fraser and Dickinson, 1973), which alter the disease progression and pathology in mice in a similar manner to human diseases. Prion strains RML and ME7 have been developed, whereby prions originally derived from sheep and goat scrapie have been serially passaged in mice resulting in mouse-adapted prions with high attack rates and well-defined incubation periods in a laboratory setting (Chandler, 1961; Zlotnik and Rennie, 1963). Mouse bioassay has been routinely used to measure prion infectivity (Sandberg et al., 2011; Sandberg et al., 2014), as well as to further understand disease modifiers in a mammalian biological system (Akhtar et al., 2013; Grizenkova et al., 2012). In a standard experiment, the time from inoculation to prion disease diagnosis (termed “incubation period”) is the defining measure.

To explore a role for *Stx6* expression in prion disease pathogenesis, we conducted a classical prion transmission study in a newly developed mouse model which has a genetic reduction in *Stx6* expression. We show that knockout of syntaxin-6 does not result in a deleterious phenotype. Following inoculation with RML or ME7 prions, *Stx6*^−/−^ mice demonstrated only a modest 12 day longer incubation period , representing an 8% and 7% increase in the incubation periods relative to wildtype controls respectively. *Stx6*^+/−^ mice showed an 8% prolongation of incubation periods following RML inoculation, although no extension in the incubation period was seen with ME7-inoculated *Stx6*^+/−^ animals. No differences in neuronal loss, spongiform change or PrP deposition were seen between the genotypes, however a variable effect of *Stx6* expression on gliosis was observed at endpoint.

## 2 Results

### 2.1 Generation of Stx6 knockout C57BL/6N mice

**Fig. 1:**
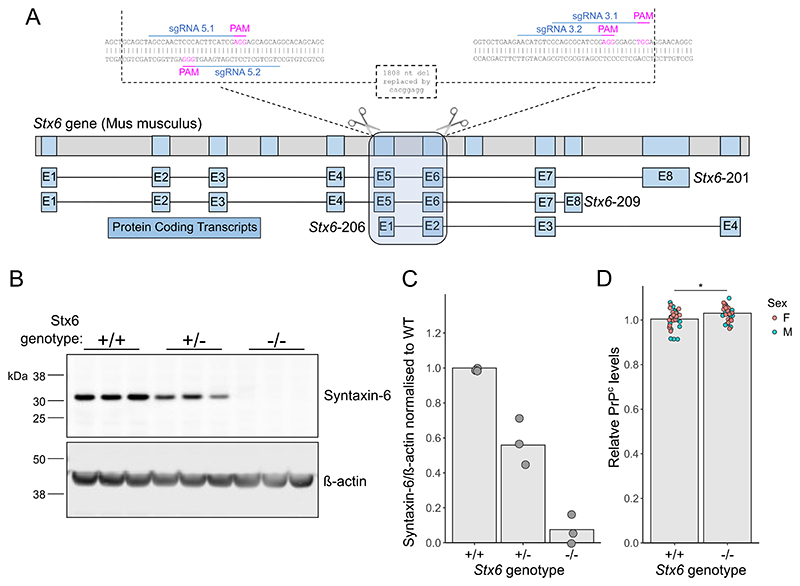
Development and validation of *Stx6* knockout mice. (A) Strategy for CRISPR/Cas9-mediated knockout of *Stx6* in C57BL/6N mice. Four single guide RNAs (sgRNAs) targeting the DNA sequences in exons 5 and 6 of the two main *Stx6* protein-coding transcripts (*Stx6*–201 and *Stx6*–209), also encompassing the N-terminal portion of the shorter *Stx6*–206 transcript, were selected for gene editing in zygotes. This resulted in an 1808 nucleotide (nt) deletion replaced by an 8 nt insertion. Top: Full *Stx6* genomic location. Bottom: protein coding transcripts. Inset: CRISPR/Cas9 genome editing at *Stx6* locus. (B) Representative quantitative immunoblots with anti-syntaxin 6 and anti-β actin antibodies of whole brain homogenates from *Stx6*^+/−^, *Stx6*^−/−^ and *Stx6*^+/+^ mice and (C) quantification of syntaxin-6 intensity relative to β-actin normalised to *Stx6*^+/+^ control demonstrates loss of the primary ~32 kDa protein isoform in *Stx6*^−/−^ mice (0.0757% ± 0. 0.0652 (mean ± SD)) with ~50% expression (55.9% ± 0. 0.131) in *Stx6*^+/−^ mice (*n* = 3/genotype, mean of 3 replicates per animal shown). (D) Analysis of PrP^C^ expression in whole brain homogenates from *Stx6*^−/−^ and *Stx6*^+/+^ early adult 9 week old mice (*n* = 30/genotype, mixed sex) by ELISA shows minor increase in relative PrP^C^ expression in *Stx6*^−/−^ mice relative to wildtype (*Stx6*^+/+^: 1.004 ± 0.0441; *Stx6*^−/−^: 1.030 ± 0.0337 (mean ± SD)). Values are relative to *Stx6*^+/+^. Relative differences were assessed by 2-way ANOVA with sex and genotype as factors.

We generated mice with homozygous deletion of *Stx6* (*Stx6*^−/−^) on the C57BL/6N background as part of the International Mouse Pheno-typing Consortium (Groza et al., 2022) (IMPC) via CRISPR/Cas9-mediated deletion of 1808 nucleotides spanning two critical exons (exons 6 and 7 of transcripts *Stx6*–*201* and *Stx6*–*209* as depicted in Fig. 1A, present within all protein coding isoforms) replaced by an 8 nucleotide insertion, generating a premature stop codon. Quantitative immunoblotting of whole brain homogenates demonstrated the loss of the primary protein product at ~32 kDa in mice with homozygous deletion of *Stx6* (*Stx6*^−/−^), whilst heterozygous mice (*Stx6*^+/−^) expressed ~50% of the protein compared to wildtype animals (*Stx6*^+/+^) (*Stx6*^−/−^: 0.0757% ± 0.0217 (mean ± SEM); *Stx6*^+/−^: 55.9% ± 0.0436) (Fig. 1B-C). This was also seen with an N-terminal syntaxin-6 antibody by immunoblotting (Supplementary Fig. 1) and we further validated knockout using immunohistochemistry as an orthogonal methodology (Supplementary Fig. 2). Reduced expression of PrP^C^ has previously been shown to extend the incubation period in mice (Büeler et al., 1993). Therefore to determine any differences associated with *Stx6* expression, brain PrP^C^ levels were assessed by enzyme-linked immunosorbent assay (ELISA). This analysis showed a very modest increase in relative PrP^C^ levels in *Stx6*^−/−^ mice compared to *Stx6*^+/+^ controls (*Stx6*^+/+^: 1.004 ± 0.0441; *Stx6*^−/−^: 1.030 ± 0.0337 (mean ± SD); *P* = 0.0150)) (Fig. 1D).

### 2.2 Stx6^−/−^ mice are physiologically normal

**Fig. 2:**
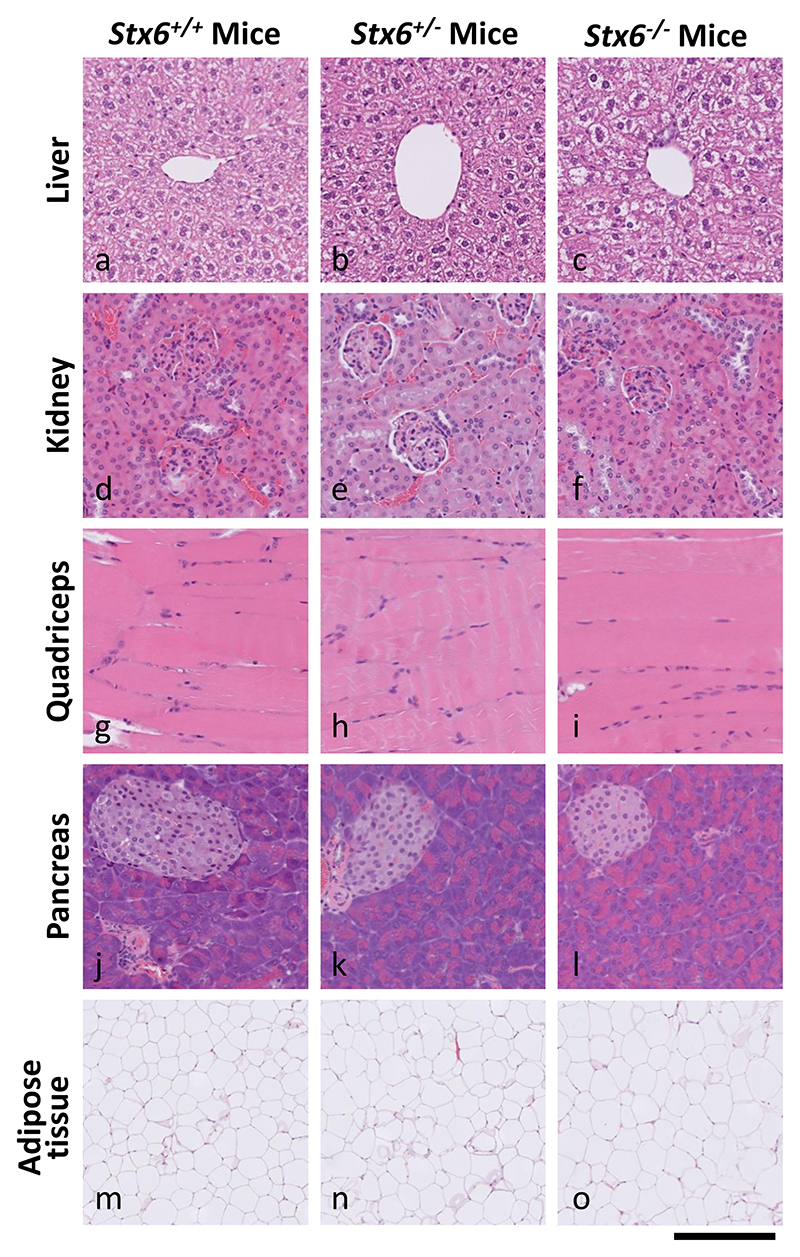
Multi-organ H&E staining shows expected tissue architecture and appearance in *Stx6*^−/−^ and *Stx6*^+/−^ mice which lack consistent tissue-intrinsic pathologies. The following organs were harvested from *Stx6*^+/+^ (*n* = 5), *Stx6*^+/ -^ (n = 5) and *Stx6*^−/−^ (*n* = 4) female mice at 56–61 weeks of age: liver, pancreas, kidney, skeletal muscle and white adipose. These were formalin fixed, processed to paraffin and underwent haematoxylin and eosin (H&E) staining, revealing no consistent differences between genotypes. Scale bar corresponds to 120 μm (a-l), and 500 μm (m-o).

To determine any gross changes in organ morphology associated with altered *Stx6* expression, haematoxylin and eosin (H&E) analysis was performed on liver, pancreas, kidney, skeletal muscle and adipose tissue dissected from 4 to 5 adult mice (4 x *Stx6*^−/−^, 5 x *Stx6*^+/−^, 5 x *Stx6*^+/+^, see Materials and methods). As shown in Fig. 2, there were no differences in the appearance and architecture between any tissue from *Stx6*^−/−^, *Stx6*^+/−^ and *Stx6*^+/+^ animals aside from moderate liver steatosis in one *Stx6* null animal, however as this was only found in one animal it is unlikely to be related to the loss of *Stx6* expression (Supplementary Fig. 3). Furthermore, analysis of skeletal structure by computed tomography (CT) scanning did not reveal any obvious differences in bone or skeleton architecture between genotypes (Supplementary Fig. 4).

Upon generation, *Stx6*^−/−^ mice were subject to the standard IMPC phenotyping program at MRC Harwell (Dickinson et al., 2016) (www.mousephenotype.org). This analysis suggested alterations in some blood clinical chemistry parameters including increased blood urea nitrogen in early adult females and increased circulating alkaline phosphatase in early adult males, but these findings were not replicated in our independent sample (*P* > 0.01) (Supplementary Table 1, Supplementary Fig. 5). In a pilot study, aged mice were examined on a range of behavioural and motor tasks, including nest building, rotarod, grip strength, pellets burrowed and weight of food consumed, with *Stx6*^−/−^ and *Stx6*^+/−^ (*n* = 5 per group) broadly comparable with wild-type animals (the study was not powered to detect subtle differences in behaviours). Overall, reduction in *Stx6* expression did not lead to a detectable deleterious phenotype in these animals.

### 2.3 Mice with genetic reduction of Stx6 have differing incubation periods when infected with two mouse-adapted prion strains

To determine the effect of *Stx6* expression on the incubation period of prion disease, *Stx6*^−/−^, *Stx6*^+/−^ and *Stx6*^+/+^ female mice were inoculated intracerebrally with two mouse-adapted scrapie prion strains, RML and ME7, or PBS as a vehicle control (Table 1).

**Fig. 3:**
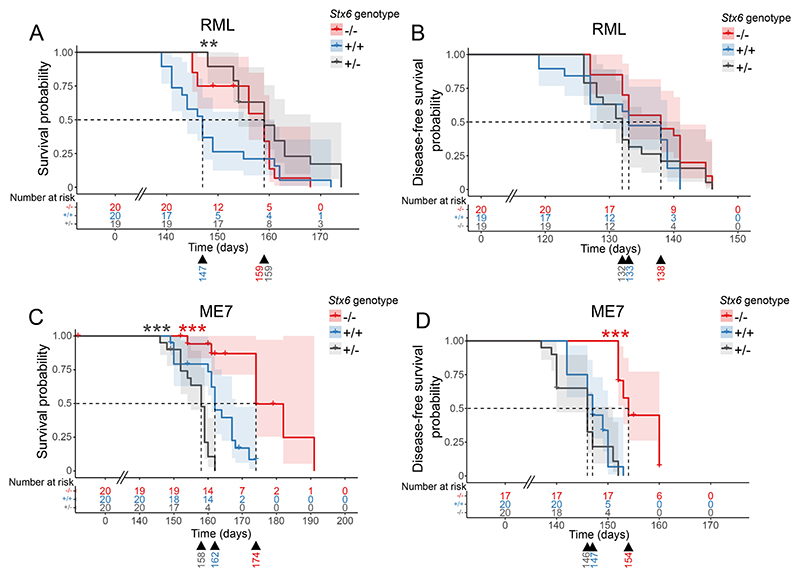
Incubation periods in mice with *Stx6* reduction following intracerebral inoculation with RML and ME7 mouse-adapted scrapie prions. (A-D) Kaplan-Meier curve of survival probability following intracerebral inoculation of *Stx6*^−/−^ (red), *Stx6*^+/−^ (grey) and *Stx6*^+/+^ (blue) C57BL/6N mice (*n* = 20 per group) with 1% brain homogenate from (A,B) RML and (C,D) ME7 infected C57BL/6N mice. (A, C) Survival probability until animals were culled due to scrapie sickness shows a 12-day increase in median incubation period for (A) *Stx6*^−/−^ and *Stx6*^+/−^ mice following RML inoculation (significant only for *Stx6*^+/−^ animals) and (C) for *Stx6*^−/−^ mice only following ME7 inoculation relative to Stx6^+/+^ mice. (B,D) Disease-free survival probability (time until onset of the first scrapie symptom) (B) does not show a significant association with *Stx6* genotype following RML-inoculation but (D) a 7-day increase in *Stx6*^−/−^ mice inoculated with ME7, relative to *Stx6*^+/+^ mice. Significance shown from pair-wise log-rank test for *Stx6*^−/−^ (red asterix) and *Stx6*^+/−^ (black asterix) mice relative to wildtype controls (** *P* > 0.01; *** *P* > 0.001). Median incubation period shown below each plot. Crosses indicate censored animals. (For interpretation of the references to colour in this figure legend, the reader is referred to the web version of this article.)

Following inoculation with RML prions, the incubation period in *Stx6*^−/−^ and *Stx6*^+/−^ mice differed by 12 days relative to wildtype animals although statistical significance was only reached for *Stx6*^+/−^ animals (*Stx6*^+/+^ = 147 days [144–161] (median [95% confidence interval]), *Stx6*^+/−^ = 159 days [154–168], *Stx6*^−/−^ = 159 days [156–160]) (Fig. 3A; Table 1). Furthermore, as a number of animals were censored from analysis due to early loss from intercurrent illness (especially for ME7 inoculated animals, where the majority of animals (13/20) were culled due to incontinence prior to full scrapie diagnosis, a likely unclassified symptom of prion diseases in mice (unpublished data); see Materials and methods, Table 1 and Supplementary Tables 4–5), the time from inoculation until onset of the first scrapie symptom was analysed (previously used as an alternative measure of incubation period (Carlson et al., 1986)). There was no significant association of *Stx6* genotype with incubation period by this measure following RML inoculation (*P* = 0.14, Fig. 3B), although there was still a modest, nominal increase in incubation period in *Stx6*^−/−^ animals compared to *Stx6*^+/+^ (*Stx6*^+/+^ =133 days [127–139] vs *Stx6*^−/−^ =138 days [133–141]; *Stx6*^+/−^ =132 days [129–141]). Although this analysis allows for greater inclusion of animals who were succumbing to the disease, identification of the first neurological symptom is typically a more variable assessment which likely underpins the differences between these two analyses. Nonetheless, a dose-dependent effect of *Stx6* expression on incubation period following inoculation with RML prions was not shown.

In ME7-inoculated animals, incubation period was also increased by 12 days with complete loss of *Stx6* compared to wildtype animals (*Stx6*^+/+^ = 162 days [161–168]) vs *Stx6*^−/−^ = 174 days [174 – NA], Fig. 3C; Table 1), however there was a 4 day decrease seen with heterozygous *Stx6* expression with this strain (*Stx6*^+/−^: 158 days [155–160]). In the analysis of time until onset of first neurological symptom there was still a significant increase in *Stx6*^−/−^ mice compared to *Stx6*^+/+^ animals of 7 days (*Stx6*^+/+^ = 147 days [146–150] vs *Stx6*^−/−^ =154 days [153–160]) (Fig. 3D), however there was no significant effect of partial *Stx6* expression loss on this prion strain in this analysis (*Stx6*^+/−^ = 146 days [140–151]), again indicating that there is no direct relationship between level of *Stx6* expression and incubation period with this strain. Although there was no clear correlation between *Stx6* expression level and incubation period, the independent, repeated observations of extended incubation period in *Stx6* knockout animals with two distinct prion strains supports a general pathological role of expression of this gene in prion disease progression which warrants further investigation.

### 2.4 Neuropathological features and Stx6 expression

**Fig. 4:**
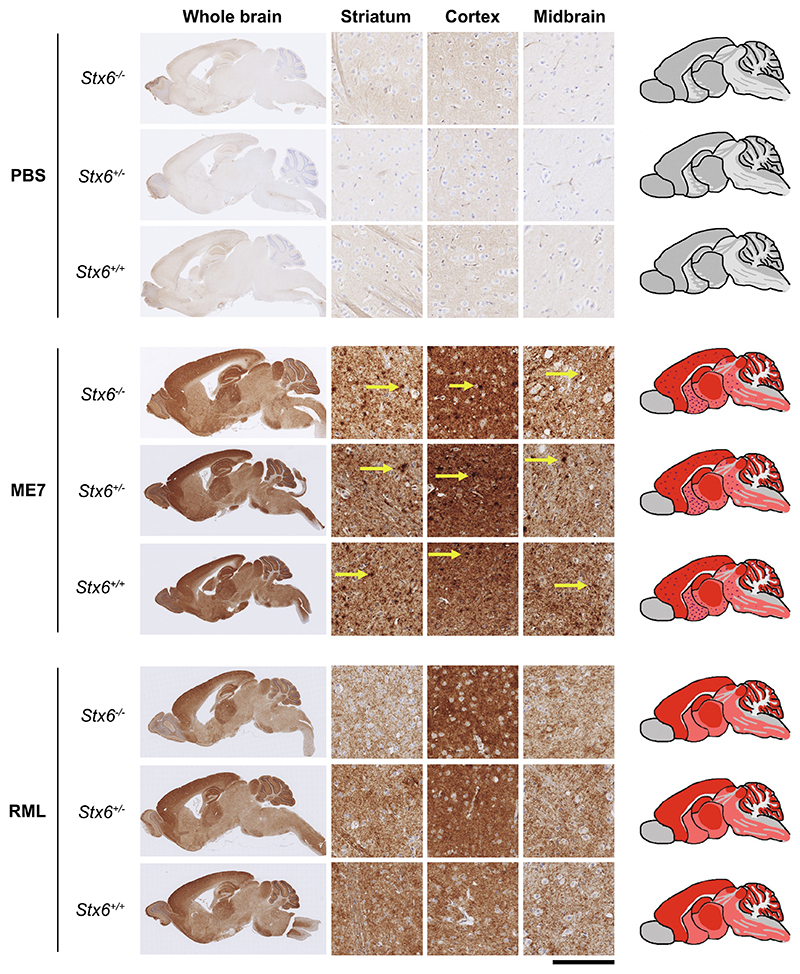
Knockout of *Stx6* does not alter PrP deposition in prion infected mice. Immunohistochemistry using anti-PrP antibody ICSM35 in whole brain (left) with schematic depiction of PrP deposition (right; pink shading: moderate PrP deposition; red shading: intense PrP deposition; red dots: PrP micro-plaques) or representative images from striatum, cortex and midbrain (middle) from *Stx6*^−/−^, *Stx6*^+/−^ and *Stx6*^+/+^ mice inoculated with PBS (top) or ME7 (middle) and RML (bottom) mouse-adapted scrapie prions, shows expected PrP deposition in all prion-inoculated samples at end-point, with widespread diffuse staining for both strains and additional presence of micro-plaques with ME7 (yellow arrows). There was no evidence of spontaneous PrP deposition in control animals or gross difference evident between genotypes. Scale bar corresponds to 100 μm in the high-power magnification and 1.8 mm for the overview images. (For interpretation of the references to colour in this figure legend, the reader is referred to the web version of this article.)

Prion disease is typically characterised by histological features of spongiform vacuolation, neuronal loss and astrocyte proliferation (Budka, 2003), along with deposition of PrP aggregates in the brain and a strong microglia response, which can be identified upon brain biopsy in patients, or on post-mortem histopathological analysis of the brain in patients and animals (Wenborn et al., 2015). Immunohistochemical staining with an anti-PrP antibody detected PrP deposits at the end-stage of the disease following intracerebral inoculation with RML and ME7 prions in all animals assessed (7–10 mice analysed per group, see Materials and methods) (Fig. 4). With both prion strains, disease-associated PrP can be detected with a diffuse staining pattern in grey matter areas. In ME7-inoculated animals there was additional widespread deposition of micro-plaques as previously described (Wenborn et al., 2015). At this resolution and time-point there was no difference evident in appearance, extent or localisation of PrP aggregates between *Stx6* genotypes.

Considering this was the first characterisation of this mouse strain, we also assessed whether manipulation of this genetic risk factor alone was sufficient to cause neuropathological changes in the absence of prion infection. Knockout of *Stx6* did not cause detectable deposition of disease-associated PrP in PBS-inoculated controls.

**Fig. 5:**
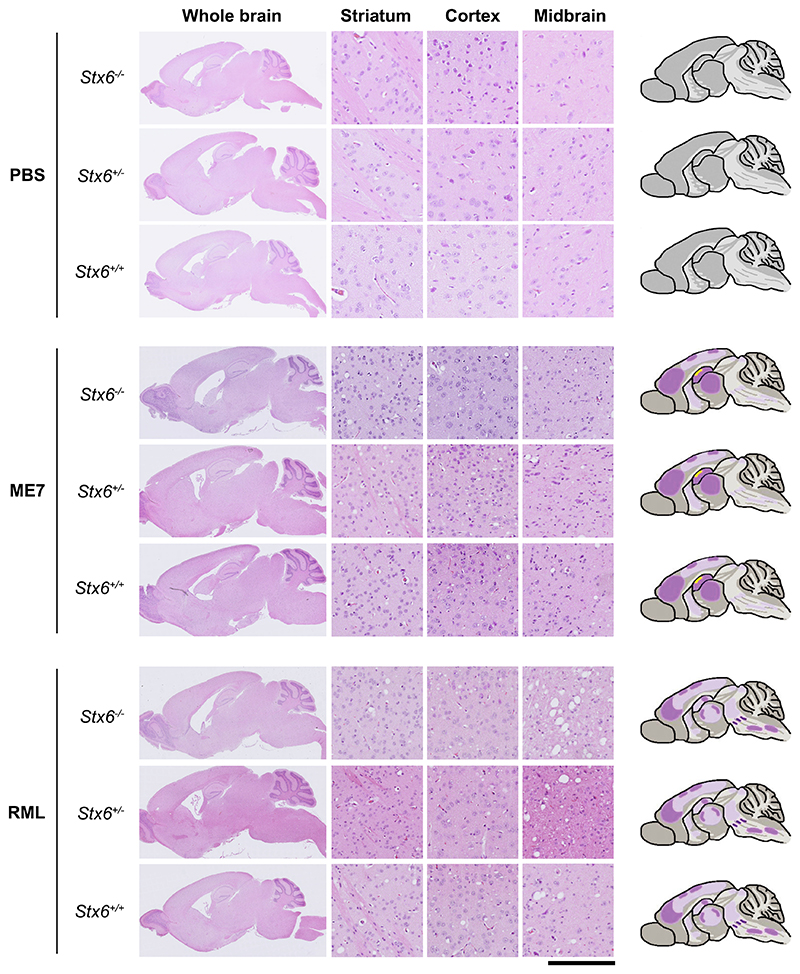
Neuronal loss and spongiform change were comparable in end-stage *Stx6*^−/−^, *Stx6*^+/−^ and *Stx6*^+/+^ mice. Haematoxylin and eosin (H&E) staining of whole brain (left) with schematic depiction of spongiosis (right; light purple shading: widely dispersed mild spongiosis; darker purple shading: moderate spongiosis; yellow shading: neuronal loss) and representative images from striatum, cortex and midbrain (middle) from *Stx6*^−/−^, *Stx6*^+/−^ and *Stx6*^+/+^ mice inoculated with PBS (top) or ME7 (middle) and RML (bottom) mouse-adapted scrapie prions shows expected spongiform pathology in all samples at end-point, including mild hippocampal neuronal loss with ME7, with no gross differences evident between genotypes. No neuropathology was evident in PBS-inoculated controls. Scale bar corresponds to 100 μm in the high-power magnification and 1.8 mm for the overview images. (For interpretation of the references to colour in this figure legend, the reader is referred to the web version of this article.)

To determine whether there were any differences in spongiform vacuolation with *Stx6* expression at endpoint, H&E staining was used to identify morphological changes in the tissue. This analysis revealed the presence of distinct small, round vacuoles within the neuropil in multiple brain regions including the striatum, cortex and midbrain in prioninoculated animals, reflecting the expected spongiform change associated with these mouse prion strains (Fig. 5). This analysis also showed the typical neuronal loss within the hippocampus following ME7 inoculation. No changes were identified in controls demonstrating knockout of *Stx6* alone does not induce pathological changes.

Taken together, these results provide some support for maintenance of prion strain type in mice with genetic reduction of syntaxin-6, with no clear differences in PrP deposition patterns and spongiform vacuolation between genotypes. To further explore whether *Stx6* exerts a selection effect for propagation of distinct prion strains, we performed western blotting on end stage brain homogenates. This demonstrated no change of PrP^Sc^ types with modified *Stx6* expression in either RML- or ME7-inoculated animals as expected (Supplementary Fig. 6). However, serial transmission would be required to confirm there is no changes in strain type, which may be undetectable using these biochemical and neuropathological methods.

### 2.5 Quantitative assessment of gliosis in prion-infected Stx6^+/+^ and Stx6^−/−^ animals

As widespread activation and proliferation of both astrocytes and microglia is a hallmark of both human and animal prion disease, which progresses with disease duration, immunohistochemical staining was performed to detect activated astrocytes and microglial cells allowing quantification of any modulatory effects *Stx6* expression may have on neuroinflammatory phenotypes in prion disease. Furthermore, control animals were analysed to determine if *Stx6* knockout alone causes any inflammatory phenotypes.

**Fig. 6:**
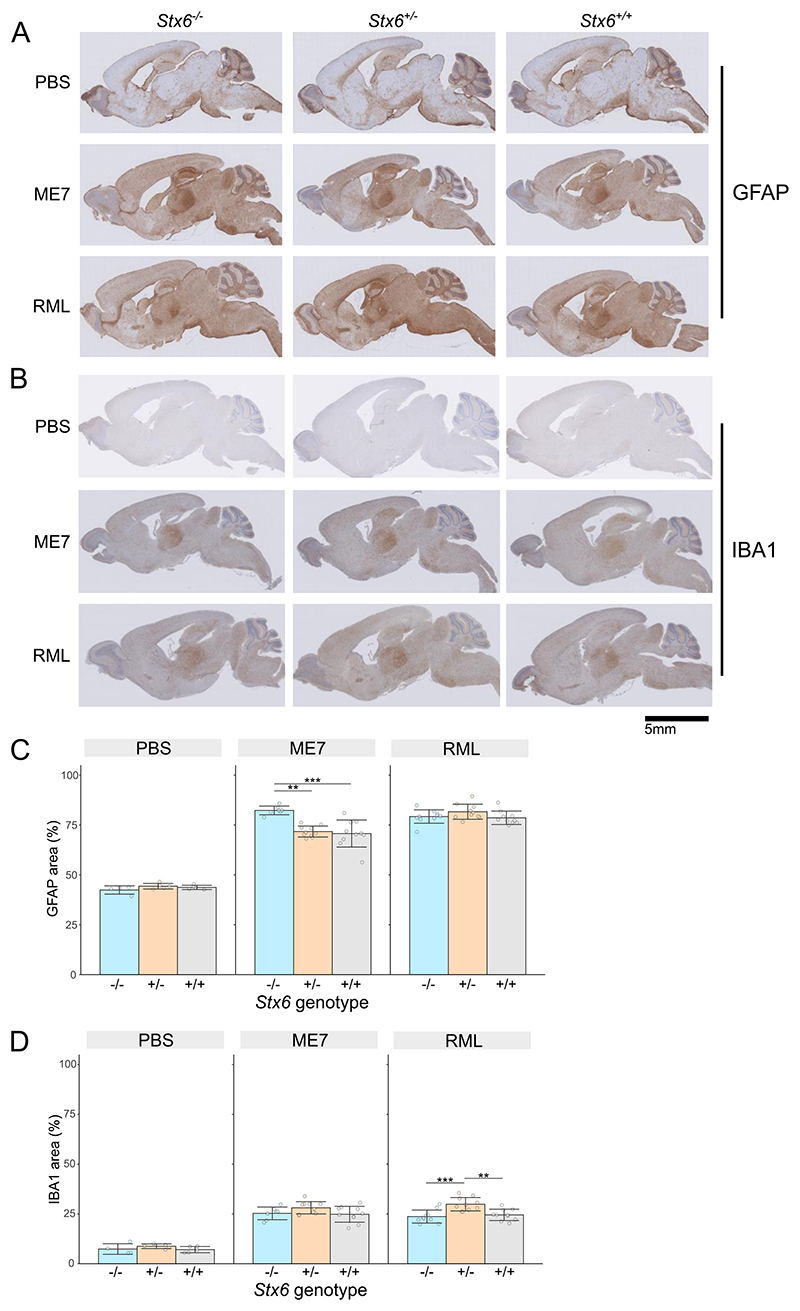
*Stx6* genotype and neuroinflammation in prion disease. (A, B) Example images of immunohistochemistry in *Stx6*^−/−^, *Stx6*^+/−^ and *Stx6*^+/+^ mice inoculated PBS control or ME7 and RML mouse-adapted scrapie prions with (A) anti-GFAP antibody to measure reactive astrocytes and (B) anti-Iba1 antibody to measure microgliosis, shows expected neuroinflammation in prion-infected animals, including expected intense staining in the thalamus with both antibodies and intense GFAP staining in the hippocampus. (C, D) Quantification of percentage area stained with (C) anti-GFAP and (D) anti-Iba1 antibody demonstrates a ~ 10% increase in astrocyte area in ME7-inoculated *Stx6*^−/−^ animals and a ~ 5% increase in microglia area in *Stx6*^+/−^ mice in RML-inoculated animals (mean ± SD; linear regression model adjusted for age of death; ** *P* > 0.01, *** *P* > 0.001).

Immunohistochemical staining of reactive astrocytes with an anti-GFAP antibody confirmed the expected widespread astrocyte proliferation, with particular intensity in the thalamus and hippocampus as previously described with these prion strains (Makarava et al., 2019) (Fig. 6A). Quantification of mean percentage area shows >70% immunoreactivity in brain tissue under all prion-inoculated conditions (ME7: *Stx6*^−/−^ = 82.3% ± 2.17 (mean ± SD), *Stx6*^+/−^ =71.7% ± 2.75, *Stx6*^+/+^= 70.7% ± 6.79; RML: *Stx6*^−/−^ =79.2% ± 3.37, *Stx6*^+/−^= 81.6% ± 3.78, *Stx6*^+/+^ =78.6% ± 3.37) compared to ~40% in PBS-inoculated animals (PBS: *Stx6*^−/−^ =42.4% ±2.08, *Stx6*^+/−^ =44.3% ± 1.41, *Stx6*^+/+^ = 43.7% ± 1.07) (Fig. 6C). This highlighted a ~ 10% increase in astrocyte area in *Stx6*^−/−^ mice inoculated with ME7 relative to both *Stx6*^+/−^ (P_adj_ = 1.70 × 10^−3^) and *Stx6*^+/+^ animals (P_adj_ = 7.57 × 10^−4^) after adjusting for age of death to take into account the later tissue collection for the *Stx6*^−/−^ mice with extended incubation periods. Of note, there was no significant association between age and GFAP area stained. However no difference was observed between *Stx6*^+/−^ mice and wildtype controls, or in RML inoculated animals, indicating there is no direct relationship between *Stx6* expression and astrocyte response. Immunostaining with an anti-Iba1 antibody to detect microgliosis demonstrated the expected visible microglia activation following inoculation with both RML and ME7 prions, occupying 25–30% of the whole brain area in all disease conditions (ME7: *Stx6*^−/−^ = 25.3% ± 3.17 (mean ± SD), *Stx6*^+/−^ = 28.1% ± 3.08, *Stx6*^+/+^ = 24.8% ± 3.99; RML: *Stx6*^−/−^ = 23.7% ± 3.26, *Stx6*^+/−^= 29.9% ± 3.36, *Stx6*^+/+^ =24.5% ± 2.84) compared to ~8% in control animals (PBS: *Stx6*^−/−^ = 7.37% ± 2.67, *Stx6*^+/−^ = 8.77% ± 1.17, *Stx6*^+/+^ = 7.09% ± 1.56) (Fig. 6B, D). In all conditions *Stx6*^+/−^ mice demonstrated a modestly higher proportion of microglia relative to both *Stx6*^+/+^ and *Stx6*^−/−^ mice, which was significant in RML inoculated animals after adjusting for different ages at death (P_adj_ = 4.07 × 10^−3^ and P_adj_ = 1.74 × 10^−4^ respectively), however there was no difference in *Stx6*^−/−^ animals relative to control or in ME7 inoculated animals, again indicating there is no clear relationship between *Stx6* expression and microgliosis in this model.

## 3 Discussion

### 3.1 Incubation period differences in mice with genetic reduction of syntaxin-6 following intracerebral inoculation with RML and ME7 mouse-adapted scrapie prions

Mouse models are a suitable way to assess the role of human risk genes as they are naturally susceptible to prion diseases, and prioninfected animals faithfully recapitulate many of the key aspects of human disease. In this study, C57BL/6N female mice with genetic reduction of syntaxin-6 showed prolonged mouse prion disease incubation periods for both prion strains tested, however this does not appear to correlate directly with syntaxin-6 expression. Furthermore, the observed extension of 12 days in prion-infected *Stx6*^−/−^ animals was modest, which may reflect the moderate effect sizes for typical risk alleles detected by GWAS (*STX6* odds ratio (OR) = 1.16) (Jones et al., 2020) or potential compensatory mechanisms associated with constitutive gene knockout. Alternatively, it remains possible that modest changes in incubation periods might have resulted from imbalance in unmeasured confounding factors with genotypes being inoculated on different days, or simply chance effects. Indeed, when groups of genetically identical FVB/N were inoculated in different groups at different times, median incubation between individual groups ranged between 103 and 137 days despite intragroup variation being very low (Tamgüney et al., 2008), substantially greater variation than what was observed between different arms of this study. The modest differences in incubation time observed therefore need cautious interpretation.

To explore whether differences in PrP^C^ levels could be the driving force of these modest differences in incubation period, we conducted a highly powered, robust study comparing PrP^C^ levels in *Stx6*^+/+^ and *Stx6*^−/−^ mice. We powered the study to detect an 8% difference in PrP^C^ levels, our quantitative suggestion of the change in PrP^C^ expression which could drive the observed difference in incubation period based on in-house data. Interestingly, this study showed a modest increase in PrP^C^, which makes this explanatory mechanism unlikely. However, we cannot exclude subtle effects on the distribution of PrP^C^ and it remains possible, even likely, that *Stx6* modifying effects are exerted through altered trafficking of PrP^C^ or disease-associated forms of the protein. Furthermore, considering the syntaxin-6 risk effect was identified for sCJD, such an effect may not be sufficient to substantially modulate incubation times in a prion-infection paradigm using 1% infected brain homogenate with the high infectious prion dose administered being substantially different from a disease-initiating event in sporadic disease. Therefore, future experiments using lower titres are warranted to explore this further.

Although we recently identified risk variants associated with sCJD at the *STX6* locus, proximity of risk SNPs to genes is suggestive but not sufficient to establish causality. Integration of GWAS variants with eQTL data provided evidence *STX6* expression is associated with human disease risk. This *in vivo* data is therefore in keeping with the hypothesis derived from GWAS discovery that increased *STX6* expression promotes prion disease pathogenesis. Importantly however, prion transmission studies model a form of acquired prion disease, raising the possibility that syntaxin-6 plays a role across different aetiologies of prion disease and across species.

### 3.2 Assessment of prion-related phenotypes

No differences in neuronal loss and the extent or distribution of PrP pathology were noted between *Stx6*^+/+^, *Stx6*^+/−^ and *Stx6*^−/−^ mice. This indicates that *Stx6* expression does not alter disease progression through a gross effect on disease pathology. However, it should be noted that this was assessed in endpoint animals where neuropathology was severe and quantification of these was not possible due to processing artefacts, which may have masked more subtle differences. Furthermore, ages of mice were not matched, as *Stx6*^−/−^ mice generally survived longer before culling than the wild-type controls.

Quantitative comparisons of gliosis indicated some minor differences across genotypes, however the lack of consistency between prion strain types and the absence of a clear correlation of *Stx6* gene expression with either microgliosis or astrogliosis, suggests there is no direct relationship between *Stx6* on either of these parameters in this model. To exclude the possibility that any differences were driven by variable ages of animals at endpoint, we included age at death in days as a covariate in the regression analysis with no significant effect found. Future dissection of time points across the prion incubation period may reveal changes in neuropathology or other readouts of disease progression.

However considering syntaxin-6 itself has been proposed as a particularly promiscuous SNARE protein with a multitude of cell-type specific functions, we cannot discount that the pathological role of syntaxin-6 is mediated through non-neuronal cell types, fitting with the increasing acceptance that prion disease pathobiology is driven by the interaction of multiple cell types and non-cell autonomous mechanisms (Tahir et al., 2020; Smith et al., 2020).

### 3.3 Targeting syntaxin-6 as a therapeutic strategy

Human genetic evidence increasingly supports successful drug development programmes (Ochoa et al., 2022). The identification and proposal of *STX6* as a risk gene through GWAS therefore provokes research about therapeutic potential, which is in part supported by this *in vivo* study. Importantly for therapeutic utility, *Stx6*^−/−^ mice are viable and fertile with no gross neurological or physiological impairments. Although the IMPC have recorded some potential metabolic and behavioural alterations in *Stx6*^−/−^ mice, these were not reproducible across life stages and gender, and were not replicated in our mouse cohort. However, the very modest extension in incubation period with full knockout of syntaxin-6 and variable effect of different prion strains indicates that partial reduction in syntaxin-6 levels would be unlikely to result in a meaningful clinical benefit in established disease. These data however do not exclude a role of syntaxin-6 is disease initiation, either in the generation of the initial prion seed or the likelihood of a small infecting dose establishing disease. This notably cannot be assessed using this acquired mouse model of prion infection. If syntaxin-6 is involved in these initial events more therapeutic promise may come from targeting syntaxin-6 prior to disease onset in at-risk individuals prior to initial seeding (applicable for inherited prion disease cases). Finally, the pleiotropic role of syntaxin-6 across multiple neurodegenerative diseases, including PSP and AD, raises the potential that *STX6*-targeting therapies could have wider applicability to other neurode-generative diseases, warranting further investigation.

### 3.4 Potential mechanistic roles of syntaxin-6 in prion disease pathology

As a key intracellular trafficking protein, it is plausible that modified syntaxin-6 function alters the transport of PrP^C^ and/or disease-associated prions throughout the cell and several possible mechanisms are plausible. Considering syntaxin-6 is thought to be involved in the recycling of proteins from endosomes (Mallard et al., 2002; Chao et al., 1999), knockout of *Stx6* could conceivably result in reduced transport of disease-associated PrP away from these structures and thus increased degradation in the lysosomal pathway, with obvious benefits for prion load. Alternatively, a similar hypothesis could be proposed for transport of toxic species in prion diseases. Recent work indicated a role of syntaxin-6 in the kinetic formation of toxic PrP structures *in vitro*, however whether a direct protein interaction occurs *in vivo* is unclear (Sangar et al., 2022). Indeed, as the effects seen in this study were only modest and do not implicate a direct relationship of syntaxin-6 expression with incubation period in this acquired prion disease model, it is perhaps likely that the role of syntaxin-6 in prion diseases predominantly acts in the first stages, for example in formation of the initial prion seed or the establishment of clinical disease. Finally, it is plausible the effect of modified syntaxin-6 function has more widespread implications for cellular function, which may indirectly affect all aspects of prion disease biology.

## 4 Conclusion

Here we have generated a novel knockout mouse model for *Stx6*, a proposed GWAS-identified risk gene for sCJD, in which we found no grossly deleterious phenotypes. A transmission study in *Stx6*^−/−^, *Stx6*^+/^ and *Stx6*^+/+^ mice challenged with two prion strains showed reduced syntaxin-6 expression is associated with a modest prolongation of prion disease incubation periods, supporting a pathological role of *Stx6* expression in prion disease pathogenesis. However, outside of a moderate effect on astrogliosis, there was no effect of *Stx6* expression on the neuropathological hallmarks of prion diseases. Syntaxin-6 appears to have pleiotropic risk effects across multiple neurodegenerative diseases including PSP and AD. Thus, this work supports further exploration of the *STX6* susceptibility mechanism, which likely has relevance across multiple neurodegenerative diseases.

## 5 Materials and methods

### 5.1 Ethical approval

Work was performed under approval and license granted by the UK Home Office (Animals (Scientific Procedures) Act 1986), which conformed to UCL institutional and Animal Research: Reporting of In Vivo Experiments (ARRIVE) guidelines. Experiments were approved by the MRC Prion Unit Animal Research Scientific Committee.

### 5.2 Mice

*Stx6*^+/−^ mice (C57BL/6NTac-*Stx6*^em1(IMPC)H^/H) were generated at MRC Harwell and crossbred to establish homozygous *Stx6*^−/−^ and *Stx6*^+/+^ lines, which were used to populate the experimental cohorts. *Stx6*^+/−^ litters were obtained through crossbreeding of these two lines.

### 5.3 Genotyping

DNA was extracted from standard ear biopsies. The presence of a 108 bp deletion in *Stx6* was determined using two PCR reactions with the following primer combinations with GoTaq G2 Hot Start Polymerase (Promega): PCR 1 forward 5′-CGATCTGTGAGACTCATCGGG and reverse 5′-GGGAGTCCTAACACCACCTTC, PCR 2 forward 5′-CCTGACTCTCTGATAGCCAC and reverse 5′-ACAAAACCAAAGCCTGCAC. PCR reactions were analysed by agarose gel electrophoresis and image capture was performed on a Universal Hood II Gel Doc System (Bio-Rad).

### 5.4 Western blot of mouse brain homogenate for syntaxin-6

Brains from mice at 61–65 days were dissected on the sagittal plane and flash frozen. Following weighing, samples were ribolysed in Dulbecco’s phosphate-buffered saline (DPBS) with ceramic homogenisation beads (Bertin Technologies) at max speed for 105 s to produce 10 and 20% homogenates which were stored at − 80 °C until use.

Homogenates were diluted in DPBS with 4× SDS sample buffer (250 mM Tris base, 40% glycerol, 8% SDS, 0.04% bromophenol blue) and boiled at 95 °C for 5 min. 35 μg of total protein was loaded onto a 4–12% (*w*/*v*) Bis-Tris polyacrylamide gel (Invitrogen) and electrophoresed before being electroblotted to a nitrocellulose membrane. Membranes were blocked in Odyssey Blocking Buffer for 1 h at room temperature (RT), before incubation with anti-syntaxin-6 (1:500 clone C34B2 (Cell Signalling; 2869S) or clone 3D10 (Abcam; Ab12370)) overnight at 4 °C. Membranes were washed with 0.05% Tween-20 in phosphate buffered saline (PBST) for 3 × 5 min with agitation and incubated with anti-β actin (1:1000 rabbit polyclonal (Sigma Aldrich; A2066) or 1:5000 mouse monoclonal (Sigma Aldrich; A5441)). After washing, membranes were probed with fluorophore-conjugated secondary antibodies (IRDye 800CW Donkey anti-rabbit IgG and IRDye 680RD goat anti-mouse IgG both at 1:4000) for 1 h at RT. Membranes were washed again and imaged with Odyssey Gel Documentation System (LI-COR; Model 9120).

### 5.5 Immunohistochemical validation of syntaxin-6 knockout

Half mouse brains were fixed in 10% buffered formal-saline, processed, paraffin wax embedded and serial sections of 5 μm nominal thickness were taken. Anti-syntaxin-6 monoclonal antibody (Cell Signalling Technology, #2869; 1:200; 6 h) was used with goat anti-rabbit secondary antibody (Abcam, #ab6720; 1:200; 1 h) and Ventana proprietary detection reagents utilizing 3,3′-diaminobenzidine tetrahydro-chloride as the chromogen (DAB Map Detection Kit; Roche Tissue Diagnostics) and superblock for 8 min. Sections were treated with Discovery SCC1 cell conditioning solution.

### 5.6 ELISA for PrP^C^ expression

Brain homogenates were initially diluted to 3.5 mg/ml total protein in PBS to a final volume of 40 μl. 5 μM AEBSF and 2.5% *w*/*v* SDS was added to each sample and boiled at 100 °C for 10 min. Samples were aliquoted and stored at -80 °C until use.

High-binding 96-well flat bottom plates were pre-coated with 100 μl of 2.5 μg/ml anti-PrP ICSM18 antibody in 0.05 M carbonate coating buffer (0.035 M NaHCO_3_, 0.015 M Na_2_CO_3_, pH 9.6) and incubated at 4 °C overnight before washing x3 with 1% PBST. Before samples were added, plates were incubated with Superblock T20 (Pierce) for 1 h 37 °C and washed again with 1% PBST.

240 μl pre-heated capture buffer (37 °C, 30 × , pH 8.4; 2% N-Lauroylsarcosyl, 2% BSA, 2% Triton X-100, 50 mM Tris base pH 8.0) was added to 8 μl sample and 50 μl added to wells in triplicate. For comparison between plates, the same reference sample is added and used for normalisation of all sample data. Plates were incubated at 37 °C for 1 h before washing x3 with 1% PBST. For detection of bound PrP, 100 μl of 1 μg/ml biotinylated anti-PrP ICSM35 antibody diluted in 1% PBST was added to each well, incubated at 37 °C for 15 min and washed as previously. 100 μl streptavidin-HRP diluted 1:10,000 in PBST was then added and incubated at 37 °C for 15 min, prior to development with QuantaBlu Fluorogenic Peroxidase Substrate Kit as per manufacturer’s instructions (Thermo Scientific). Plates were quantified using a Tecan Infinite M200 plate reader with 313 nm and 398 nm excitation and emission wavelengths respectively.

Relative differences in PrP^C^ levels were assessed by 2-way ANOVA with sex and genotype as factors. This was followed by planned comparisons of the predicted means to compare the levels of the Genotype factor, Snedecor and Cochran (1989).

### 5.7 Inoculation study

Anaesthetised female *Stx6*^−/−^, *Stx6*^+/−^ and *Stx6*^+/+^ mice were inoculated intracerebrally in the right parietal lobe with 30 μl 1% (*w*/*v*) C57BL/6 mouse-adapted RML or ME7 prion-infected brain homogenate at 6–8 weeks of age (*n* = 20/group), as previously described (O’Shea et al., 2008), or with PBS (*n* = 5/group). Experimental arms were inoculated on different days. Each individual mouse was considered an experimental unit. Group sizes of 20 mice per experimental condition were used to provide sufficient power to detect a 5% difference in incubation periods, calculated assuming an approximate minimum incubation period of 150 days with a standard deviation of 7.5 days estimated from previously published studies with C57BL/6 mice (Akhtar et al., 2013; Akhtar et al., 2011), whilst allowing for loss due to incurrent illness (as expected from previous experiments of similar length (unpublished data)). Animals were allocated to groups based on genotypes. The animal technicians tasked with diagnosing scrapie sickness were blind to study design.

Mice were monitored daily and culled following diagnosis with scrapie sickness according to established criteria or identification of distinct health concerns (O’Shea et al., 2008). Incubation period was defined as the number of days from inoculation until definite scrapie diagnosis (O’Shea et al., 2008), with animals censored when culled due to other reasons (health concerns/adverse events outlined a priori in the Project license) or found dead (Table 1). Animals culled or found dead prior to inoculation were excluded from this analysis due to missingness.

Disease-free incubation period was defined as the number of days from inoculation to onset of the first scrapie-associated neurological symptom, with animals censored in the previous analysis due to wet genital region included in this analysis as a previously reported symptom of scrapie sickness, shown to have the same level of neuropathology and PrP^Sc^ deposition as mice diagnosed with scrapie sickness (data unpublished). Mice culled due to other health concerns not known to be related to prion disease remained censored from this analysis (Table 1). Animals inoculated but culled prior to onset of any neurological symptoms were excluded from this analysis due to missingness.

Kaplan-Meier survival curves and analyses were generated using RStudio packages “survival” and “survminer” and survival differences analysed using a log-rank test.

### 5.8 Western blot for prion strain typing

Following culling, brains were removed, dissected on the sagittal plane with one hemisphere flash frozen and stored at -80 °C until brain homogenate preparation. 20% (*w*/*v*) homogenates were prepared in DPBS by ribolysing with ceramic homogenisation beads (Fisherbrand Zirconium Ceramic Oxide Bulk Beads) at 6500 rpm for 45 s using the Hybaid Riboylser. 20% homogenates were stored at -80 °C until use. 20 μL aliquots of 10% (w/v) brain homogenates were prepared in DPBS. 1 μL benzonase was added and left to incubate for 10 min at RT followed by 1 h incubation with proteinase K (PK; 50 μg/ml or 100 μg/ml final concentration for ME7 and RML respectively) at 37 °C with agitation (800 rpm).

Samples were mixed with 2× Lithium Dodecyl Sulphate Sample Buffer (2× dilution NuPAGE™ LDS Sample Buffer (4×) and 5× dilution NuPAGE™ Sample Reducing Agent (10×)) with 4-(2-Aminoethyl) benzenesulfonyl fluoride hydrochloride (AEBSF) (4 mM final concentration). Samples were immediately transferred to a 100 °C heating block for 10 min. Following a 1 min spin at 21,000 x g, electrophoresis was performed on 12% (w/v) bis-tris gels (Invitrogen) with SeeBlue Prestained Molecular Weight Marker prior to electroblotting to Immobilon PVDF membrane (Millipore).

Membranes were blocked in PBST with 5% (w/v) non-fat dried skimmed milk powder and then probed with ICSM35 anti-PrP antibody (D-Gen Ltd.; 1:5000 for RML) or with 6D11 anti-PrP antibody (Bio-Legend (808004); 1:5000 for ME7) in PBST overnight. After washing (3 × 5 min followed by 3 × 15 min) the membranes were probed with a 1:10,000 dilution of alkaline-phosphatase-conjugated goat anti-mouse IgG secondary antibody (Sigma-Aldrich (A2179)) in PBST. After washing (1 h with PBST as before and 2 × 5 min with 20 mM Tris pH 9.8 containing 1 mM MgCl_2_) blots were incubated for 5 min in chemilumi-nescent substrate (CDP-Star; Tropix Inc) and visualized on Biomax MR film (Kodak).

### 5.9 Immunohistochemistry of prion-related neuropathology

Immunohistochemistry was performed as previously described with modifications (Wadsworth et al., 2021). Half mouse brains were fixed in 10% buffered formal-saline, processed, paraffin wax embedded and serial sections of 5 μm nominal thickness were taken. Deparaffinised sections were investigated for abnormal PrP, microgliosis and astrocytosis on the Ventana Discovery XT automated IHC staining machine (Roche Tissue Diagnostics) as described in (Wadsworth et al., 2017). In the PBS inoculated controls, 4 x *Stx6*^−/−^, 5 x *Stx6*^+/−^ and 5 x *Stx6*^+/+^ animals were analysed (due to one *Stx6*^−/−^ mouse lost due to incurrent illness). In the RML inoculated groups, 10 mice were analysed for each genotype. In the ME7 inoculated groups, 10 mice were analysed for *Stx6*^+/+^ and *Stx6*^+/−^ groups and 7 mice in the *Stx6*^−/−^ group (only mice culled due to scrapie sickness were analysed for histology). All 20 mice were analysed for neuronal loss in each group.

Anti-PrP monoclonal antibody ICSM35 (D-Gen Ltd) was used with biotinylated polyclonal rabbit anti-mouse immunoglobulin secondary antibodies (Dako; Agilent) and Ventana proprietary detection reagents utilizing 3,3′-diaminobenzidine tetrahydrochloride as the chromogen (DAB Map Detection Kit; Roche Tissue Diagnostics). Sections were treated with Discovery CC1 cell conditioning solution at 95 °C for 60 min followed by a low concentration of protease (Protease 3) for 12 min prior to staining.

Anti-iba1 microglial antibody (AlphaLaboratories) or anti-GFAP antibody (Agilent) were used with biotinylated polyclonal goat anti-rabbit immunoglobulin secondary antibodies (Dako; Agilent) and Ventana proprietary detection reagents (DAB Map Detection Kit). Sections were treated with Discovery CC1 cell conditioning solution at 95 °C for 60 min or a medium concentration of protease (Protease 1) for 4 min respectively prior to staining.

Conventional methods on a Gemini AS Automated Slide Stainer were used for haematoxylin staining. Positive staining controls for the staining technique were used throughout. Slides were digitally scanned on Hamamatsu NanoZoomer 360, images captured from the NDP.serve3 (NanoZoomer Digital Pathology) or NZConnect software and composed with Adobe Photoshop.

### 5.10 Immunohistochemistry quantification and statistical analysis

GFAP and Iba1 immunostaining in whole brain sections was quantified using QuPath (v0.3.0) software (4–5 PBS-inoculated mice; 7–10 prion-inoculated mice). Colour deconvolution was performed to distinguish DAB staining from haematoxylin background and pixel classification used to select tissue for analysis (whole brain). Pixel classification selected regions positive for DAB staining (classifier trained on 10% total images). Area positive for DAB staining was calculated for each image and % area stained calculated relative to total tissue area analysed. Statistical differences were determined using a linear regression model with age at death included as a confounding variable in RStudio (v1.1.463) software.

### 5.11 Haematoxylin and eosin (H&E) staining of peripheral organs

The following organs were harvested from PBS-inoculated wildtype *Stx6*^+/+^, *Stx6*^+/−^ and *Stx6*^−/−^ female mice at 56–61 weeks of age: liver, pancreas, kidney, skeletal muscle and adipose tissue. Organs were fixed in 10% buffered formal-saline and processed for haematoxylin staining as previously described. Tissues were visually assessed for expected architecture integrity and for vacuolation in a semi-quantitative manner.

### 5.12 Computer tomography (CT) scanning

*Stx6*^+/+^ (*n* = 2) and *Stx6*^−/−^ (n = 2) male mice were culled at 12 weeks of age and frozen at − 20 °C until CT scanning. Bone abnormalities were assessed using the Quantum x2 microCT scanner. Analysis was done on Analyse 14.0 software.

### 5.13 Clinical chemistry analysis

Terminal blood was collected from 100 day old *Stx6*^+/+^ and *Stx6*^−/−^ animals by post-mortem cardiac puncture. Serum was prepared by allowing clotting for 10 mins followed by centrifugation at 2600 *g* at 4 °C for 12 min. Serum was stored in polypropylene tubes at −80 °C prior to assessment of blood metabolites using the AU680 Analyser.

## Supplementary Material

Supp. Table 1, Supp. Fig. 1-7

Supp. Table 2-7

## Figures and Tables

**Table 1:** Summary of analysis for association of *Stx6* genotype with disease incubation periods following inoculation with RML and ME7 prions.

		P*	Group	N start	N events	Median (days)	Lower 95% CI	Upper 95% CI		Diff. (vs WT) (days)	P (vs WT)**
			*WT*	*20*	*19*	*147*	*144*	*161*			
	RML	*P* = 0.0049	HET	19	18	159	154	168		+12	0.0047
Scrapie Clinical Diagnosis			KO	20	16	159	156	160		+12	0.17
			*WT*	*20*	*16*	*162*	*161*	*168*			
	ME7	*P <* 0.0001	HET	20	19	158	155	160		−4	0.00040
			KO	20	7	174	174	NA		+12	0.00075
			*WT*	*19*	*19*	*133*	*127*	*139*			
	RML	P = 0.14	HET	19	19	132	129	141		−1	0.86
First Scrapie Symptom			KO	20	20	138	133	141		+5	0.16
			*WT*	*20*	*18*	*147*	*146*	*150*			
	ME7	P *<* 0.0001	HET	20	19	146	140	151		−1	0.29
			KO	17	14	154	153	160		+7	1.20 × 10^−7^

Summary statistics for incubation periods measured as time until animals were culled once diagnostic clinical signs were noted (top) or onset of first scrapie-associated symptom (bottom) following intracerebral inoculation with 1% RML or ME7 infected C57BL/6 brain homogenate by Stx6 genotype (KO: ^−/−^; WT: ^+/+^; HET ^+/−^). Median incubation period in days listed with corresponding 95% confidence intervals. * P-value from overall log-rank test of Stx6 genotype with disease incubation period. ** P-value from a pairwise log-rank test for association of each group with relative WT controls. In the Scrapie Clinical Diagnosis analysis, animals inoculated but culled prior to scrapie sickness diagnosis were censored. In the First Scrapie Symptom analysis, animals culled prior to the onset of neurological symptoms were excluded and those culled due to other unrelated health reasons following description of a neurological symptom were censored.

## Data Availability

All data is available in Supplementary Table S3-S8. Original images of Western blots are available in Supplementary Fig. 7.

## Abbreviations

sCJD: sporadic Creutzfeldt-Jakob disease
PrP^C^: cellular prion protein
PSP: progressive supranuclear palsy
AD: Alzheimer’s disease
SNARE: soluble N-ethylmaleimide-sensitive factor attachment protein receptor
eQTL: expression quantitative trait loci
IMPC: International Mouse Phenotyping Consortium
GWAS: genome wide association study
CT: computed tomography
OR: odds ratio.
