# Supplementary material for: Characterisation and prion transmission study in mice with genetic reduction of sporadic Creutzfeldt-Jakob disease risk gene Stx6
Source: Neurobiol Dis. Author manuscript; Available in PMC 2024 Feb 6. (PMC7615600; doi:10.1016/j.nbd.2023.106363)
Supplement: Supp. Table 1, Supp. Fig. 1-7 [file EMS193748-supplement-Supp__Table_1__Supp__Fig__1_7.docx]

## Supplementary Material

### Supplementary Tables

**Supplementary Table 1:** **Significant phenotypes identified in Stx6^-/-^ mice include multiple metabolic parameters.**

| **Significant phenotype** | **Procedure** | **Parameter** | **P-value** | **Significant life stage** | **Significant sex** | **P-Value Other Gender** | **P-Value Other Life Stage** |
| --- | --- | --- | --- | --- | --- | --- | --- |
| Increased circulating alkaline phosphatase | Clinical Chemistry | Alkaline phosphatase | 6.92 x 10^-7^ | Early adult | Male | 1.24 x 10^-4^ | 0.19 |
| Increased blood urea nitrogen | Clinical Chemistry | Urea (Blood Urea Nitrogen - BUN) | 9.38 x 10^-21^ | Early adult | Female | 0.000164 | 2.97x10^-2^ |
| Increased circulating cholesterol | Clinical Chemistry | Total cholesterol | 1.48 x 10^-6^ | Late adult | Male | 0.190 | 0.54 |
| Abnormal vibrissae morphology | Combined SHIRPA and Dysmorphology | Vibrissae - appearance | 6.84 x 10^-5^ | Early adult | Male | 1 | 0.25 |
| Abnormal gait | Combined SHIRPA and Dysmorphology | Gait | 6.38 x 10^-5^ | Early adult | Both | N/A | 0.55 |
| Hyperactivity | Open Field | Periphery (P) resting time and whole arena (WA) resting time | 4.27 x 10^-5^ (P)  8.82 x 10^-6^ (WA) | Early adult | Female | 0.00573 (P)  0.000163 (WA) | 1.93x10^-3^ (P)  0.15 (WA) |
| Decreased anxiety related response | Open Field | Percentage centre movement time | 7.83 x 10^-5^ | Late adult | Female | 0.132 | 5.41 x 10^-4^ |

Phenotyping tests were performed by MRC Harwell as part of the IMPC including morphological, physiological and behavioural measurements measured at early adult (week 9-16) and late adult (week 52-59) life stages. Differences between Stx6^-/-^ mice (Stx6^em1(IMPC)H^) and wildtype C57BL6/N mice from the same facility analysed using a linear mixed model (continuous data) or Fisher’s exact test (categorical data). Listed are all associated phenotypes below the P < 10^-4^ threshold. Data and analyses downloaded from www.mousephenotype.org. Data accessed 9^th^ January 2023.

###
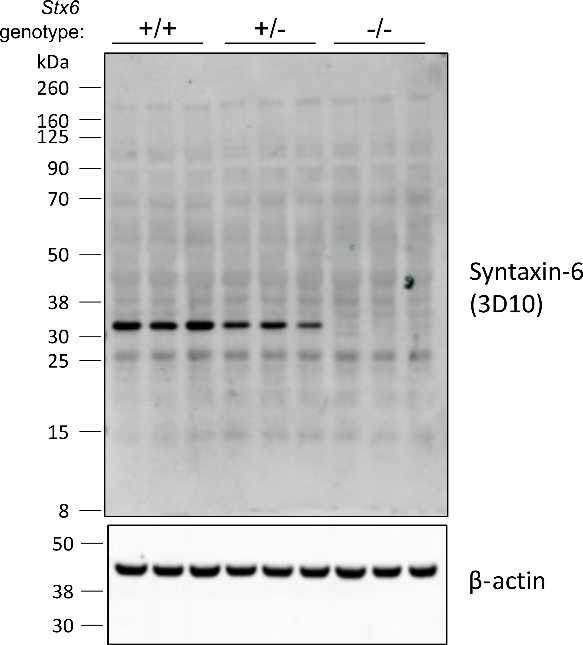
Supplementary Figures

**Supplementary Fig 1: Stx6 expression in whole brain homogenate with additional anti-syntaxin-6 antibody.** Quantitative immunoblot of whole brain homogenates from Stx6^+/-^ and Stx6^-/-^ mice relative to wildtype Stx6^+/+^ littermate controls demonstrates loss of primary ~32 kDa protein isoform in Stx6^-/-^ mice with ~50% expression in Stx6^+/-^ mice.


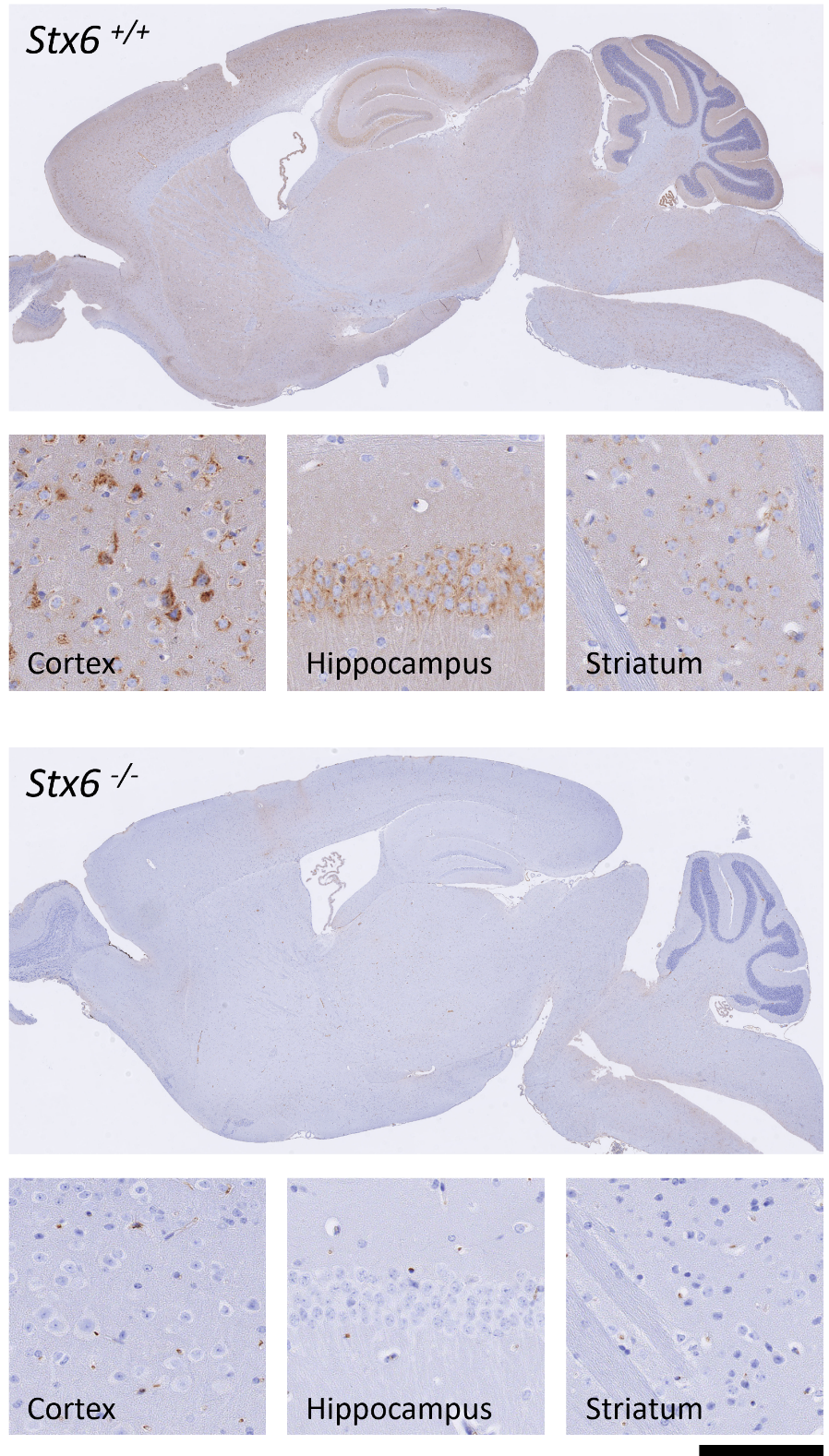


**Supplementary Fig 2: Immunohistochemical analysis of Stx6 expression in Stx6^+/+^ and Stx6^-/-^ mouse brain.** Representative images from immunohistochemistry with anti-syntaxin-6 antibody in whole brain of Stx6^+/+^ (top) and Stx6^-/-^ (bottom) mice, with example staining of cortex, hippocampus and striatum shown. Scale bar: high power 100 μm, low power 1.5 mm.


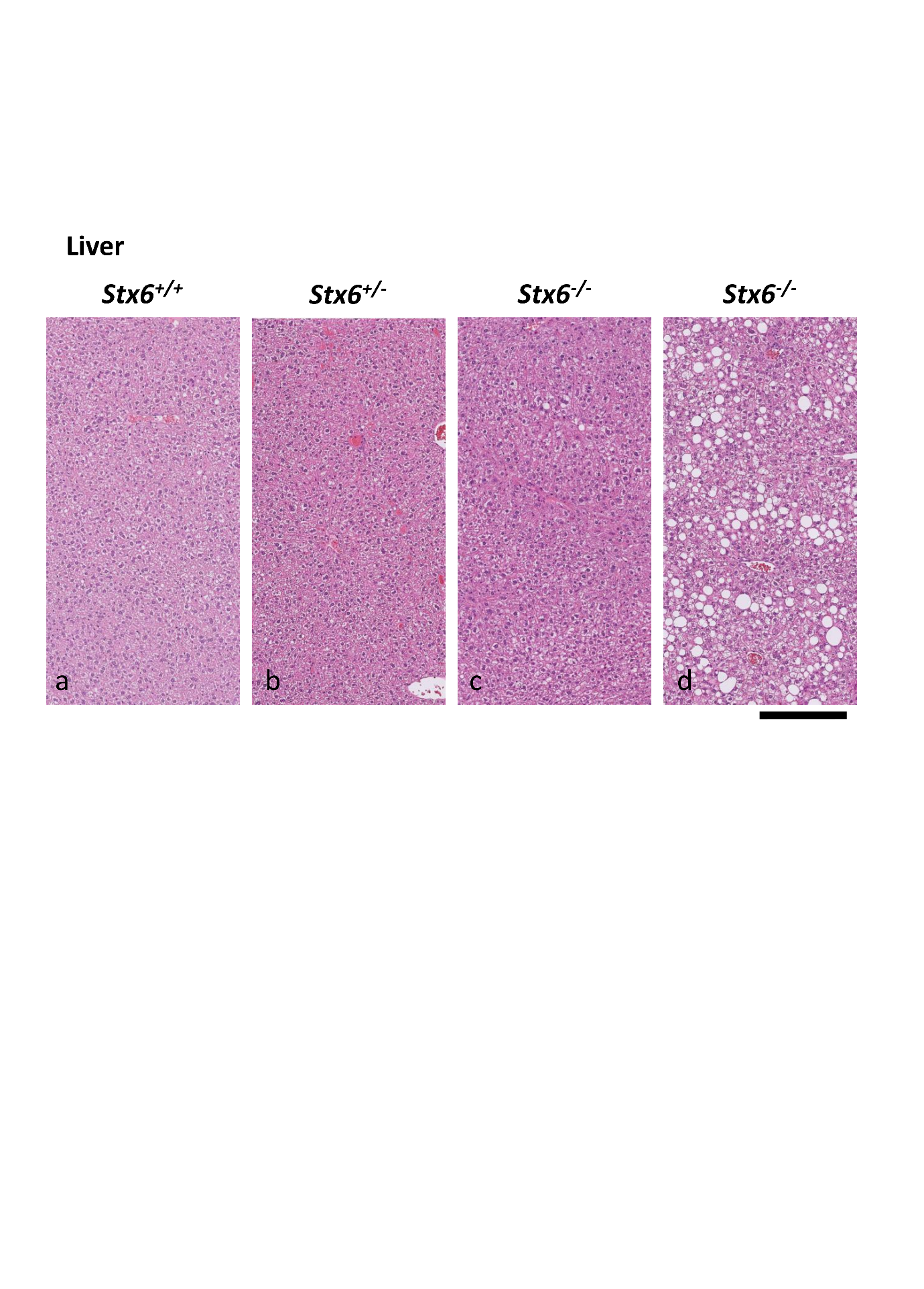


**Supplementary Fig 3: Moderate liver steatosis in one Stx6-/- animal.** H&E staining of liver from Stx6^+/+^, Stx6^+/-^ and Stx6^-/-^ mice (n = 4-5 per group; a-c) shows the expected appearance in all animals except moderate steatosis in one Stx6^-/-^ animal (d). Scale bar corresponds to 120 µm.


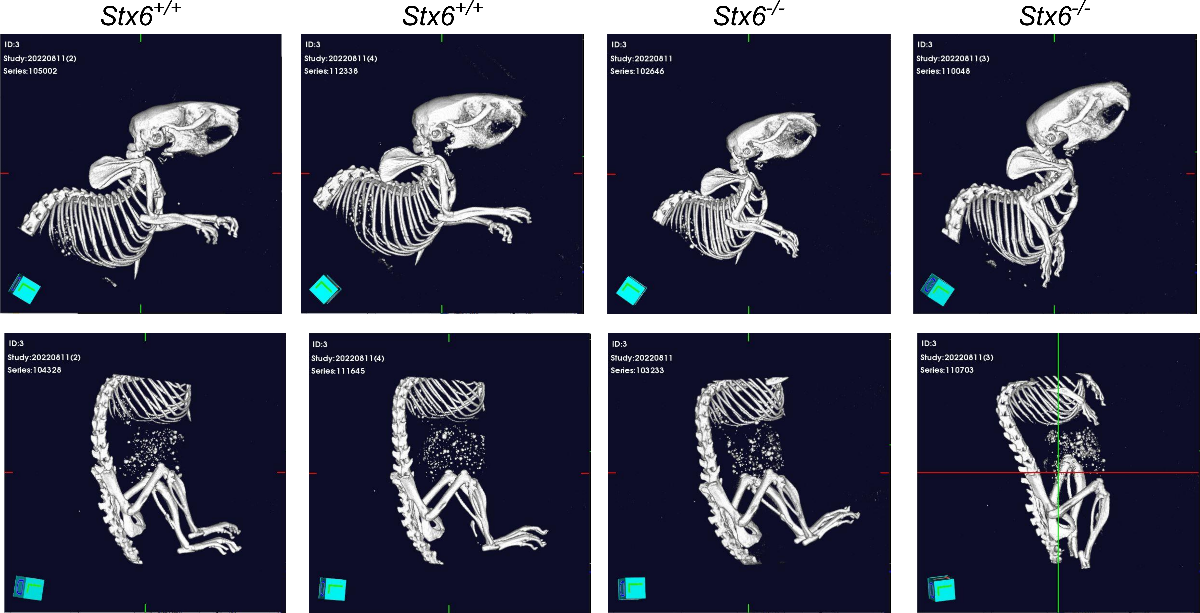


**Supplementary Fig 4. No obvious differences in skeletal structure in Stx6^-/-^ mice compared to Stx6^+/+^ mice.** Bones of Stx6^+/+^ and Stx6^-/-^ cadavers (n = 2/genotype; 3 months of age) were assessed by Computed Tomography (CT) scanning. Upper body (top) and lower body (bottom) were scanned separately.


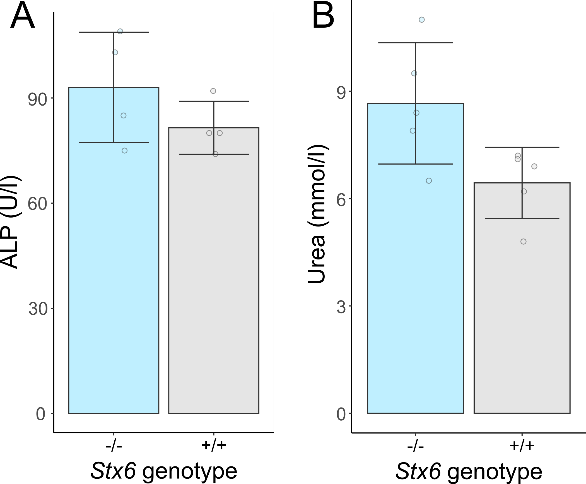


**Supplementary Fig 5: Validation of clinical chemistry phenotypes in early adult Stx6 knockout mice.** Analysis of significantly altered clinical chemistry parameters from IMPC analysis in serum of 100-day old (early adult) Stx6^-/-^ and Stx6^+/+^ mice for levels of (**A**) alkaline phosphatase (males) (ALP) and (**B**) urea (females) all P > 0.01 (Student’s t-test) (mean ± SD).


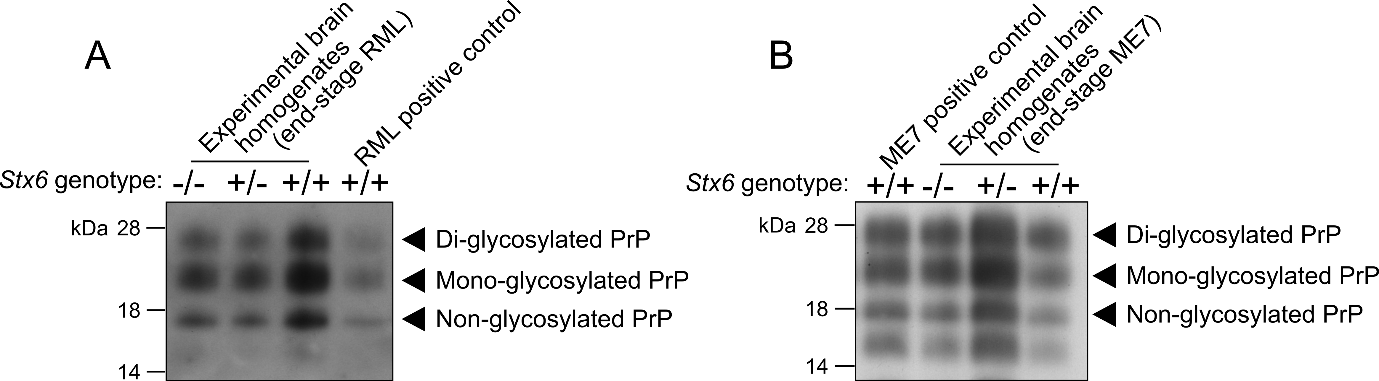


**Supplementary Fig 6: No effect of Stx6 expression on end-stage RML or ME7 prion strain type.** Western blot analysis of PK-resistant PrP in brain homogenates from (**A**) RML and (**B**) ME7-inoculated animals at disease end-stage shows expected electrophoretic mobility and glycosylation pattern of RML and ME7 inoculum respectively with all Stx6 genotypes.

**
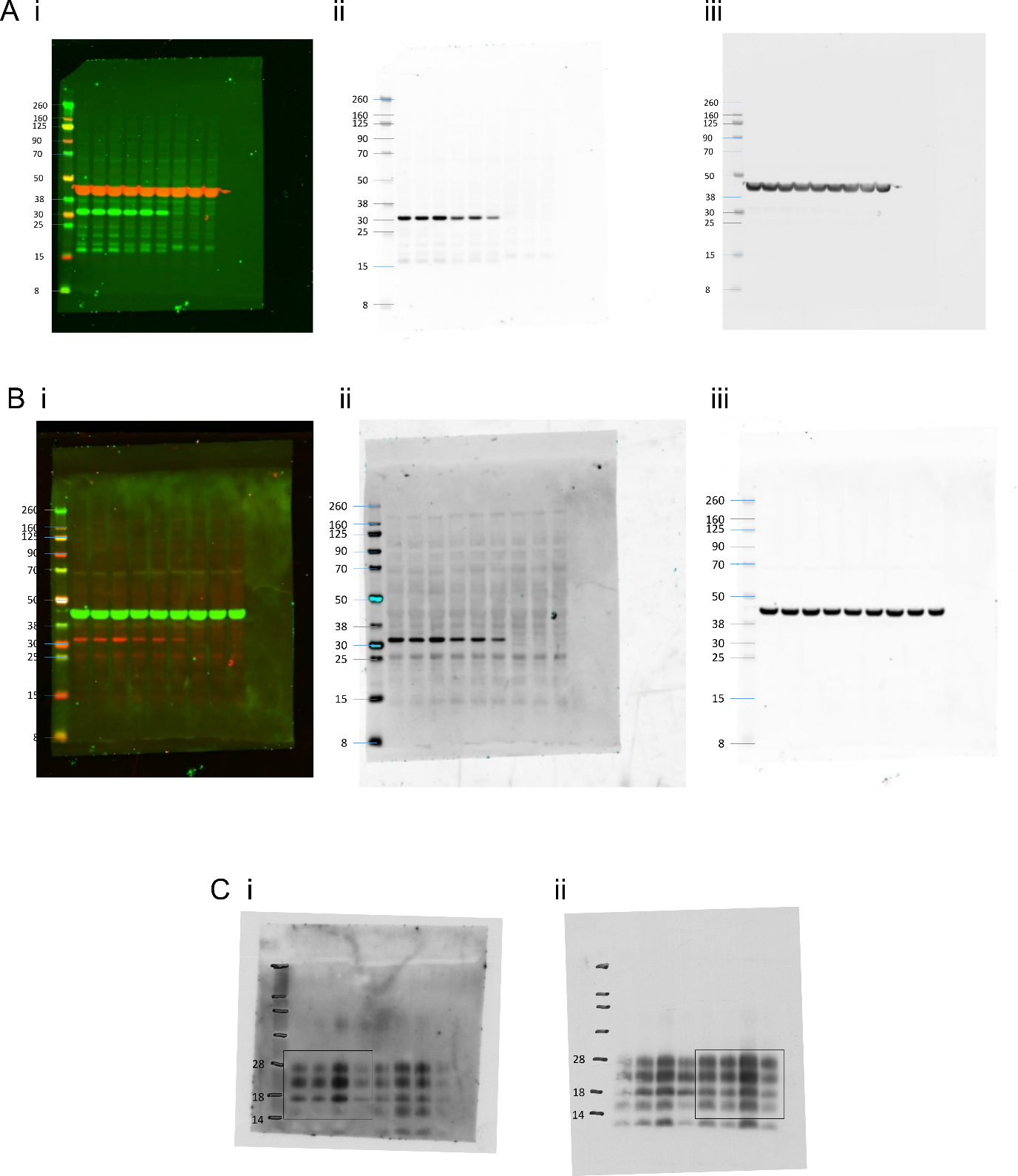
**

**Supplementary Fig 7: Original Western blot images.** Original images of Western blots used in (**A**) Figure 1 ((**i**) dual colour image, (**ii**) anti-syntaxin-6, (**iii**) anti-β actin), (**B**) Supplementary Figure 1 ((**i**) dual colour image, (**ii**) anti-syntaxin-6, (**iii**) anti-β actin) and (**C**) Supplementary Figure 5 ((**i**) RML, (**ii**) ME7, boxes indicate cropped area).
